# Generalized Strength Prediction Model for Timber Beams Strengthened Using NSM FRP Bars and FRP Sheets

**DOI:** 10.3390/polym18141705

**Published:** 2026-07-10

**Authors:** Husain Abbas, Nadeem A. Siddiqui, Mohammed S. Shaik, Tarek Almusallam, Yousef Al-Salloum

**Affiliations:** Chair of Research and Studies in Strengthening and Rehabilitation of Structures, Department of Civil Engineering, College of Engineering, King Saud University, P.O. Box 800, Riyadh 11421, Saudi Arabia; habbas@ksu.edu.sa (H.A.); nadeem@ksu.edu.sa (N.A.S.); msaleemshaik@yahoo.com (M.S.S.); musallam@ksu.edu.sa (T.A.)

**Keywords:** structural timber beams, strength prediction, near-surface-mounted (NSM) technique, FRP strengthening, hybrid reinforcement, CFRP/GFRP, analytical modeling, bond behavior, structural retrofitting, composite strengthening

## Abstract

Existing analytical models for Fiber-Reinforced Polymer (FRP)-strengthened timber beams are generally limited to individual strengthening techniques and cannot readily accommodate hybrid reinforcement systems. This study develops a generalized analytical model to predict the flexural capacity of timber beams strengthened with near-surface-mounted (NSM) FRP bars, externally bonded FRP sheets, or their hybrid combination within a unified theoretical framework. The model is formulated based on internal force equilibrium and strain compatibility, incorporating a constitutive model for timber with linear elastic tensile behavior and a bilinear compressive stress–strain relationship including post-peak softening. The generalized formulation can be readily adapted to different strengthening configurations through appropriate simplifications. The proposed model was validated against experimental results obtained from four-point bending tests on small-scale timber beams strengthened with NSM GFRP bars and externally bonded GFRP sheets. The analytical predictions showed good agreement with the experimental results, with differences generally ranging from 2% to 23%, demonstrating satisfactory predictive accuracy. The experimental results further showed that the hybrid strengthening system increased the flexural capacity of the timber beams by up to 84% compared with the unstrengthened control beams, while also improving stiffness, ductility, and overall structural response. Failure was primarily due to timber tensile rupture and longitudinal splitting, whereas the GFRP reinforcement remained effective without rupture, indicating efficient utilization of the strengthening system. The proposed generalized analytical model provides a practical and reliable design tool for predicting the flexural strength of timber beams strengthened with various FRP reinforcement configurations, thereby supporting the structural rehabilitation and sustainable retrofitting of timber structures.

## 1. Introduction

Timber has been widely used as a structural material for centuries due to its sustainability, availability, favorable strength-to-weight ratio, and aesthetic appeal. It remains a preferred choice in residential, commercial, and heritage structures. However, timber elements are vulnerable to environmental exposure, biological degradation, aging, and mechanical damage, which can significantly reduce their load-carrying capacity over time. These issues are particularly critical in flexural members, where deterioration can lead to reduced stiffness, excessive deflection, and premature failure. Consequently, strengthening and retrofitting of timber beams have become essential to extend service life and meet modern safety requirements. Recent research has therefore focused on developing efficient strengthening techniques that enhance structural performance while preserving the original geometry and integrity of timber members [1,2,3].

In addition to conventional strengthening approaches, polymer-based materials have increasingly contributed to the development of advanced timber repair and reinforcement systems. Polymer adhesives, particularly epoxy-based systems, are widely used in timber engineering due to their high bonding strength, good compatibility with lignocellulosic substrates, and ability to form durable adhesive interfaces [4,5]. The performance of these systems depends on polymer curing behavior, crosslink density, and the development of an effective polymer–wood interphase, in which chemical interactions, adhesive penetration, and mechanical interlocking govern stress transfer between the reinforcement and the timber substrate [6,7].

### 1.1. Conventional Strengthening and Repair Techniques

Traditional strengthening methods for timber beams include the use of metallic elements, additional wooden members, and epoxy-based repairs [1]. For tension members, metallic tie rods or wooden cover plates are commonly used, depending on whether unloading is possible. For bending members, local strengthening is typically achieved using metallic plates and bolts to control crack propagation, while transverse cracks are addressed using cover plates or metallic profiles.

Strengthening can also be achieved by increasing the cross-section through additional timber, steel, or concrete elements, or by inserting reinforcement within the beam while maintaining the original dimensions. External tie rods are also used to improve structural behavior. Although effective, these methods increase weight, alter geometry, and may compromise aesthetics or durability [1,2].

### 1.2. Strengthening Using Externally Bonded FRP

Fiber-reinforced polymer (FRP) composites, consisting of high-strength fibers embedded in a polymer matrix, have become a widely adopted technique for enhancing the performance of timber beams owing to their superior tensile capacity, lightweight characteristics, and excellent corrosion resistance. Externally bonded reinforcement (EBR) systems, where FRP sheets or laminates are bonded to the tension face, have been widely studied and shown to significantly enhance flexural capacity and stiffness [8,9].

Andre and Kliger [10] reviewed various strengthening configurations, demonstrating that both externally bonded sheets and near-surface-mounted (NSM) reinforcement improve structural performance. Strength increases of 40–70% and stiffness improvements up to 30% have been reported. NSM reinforcement, in particular, shifts failure toward more ductile compression-controlled modes and provides improved bond performance.

Numerous studies [9,11,12,13,14] have confirmed that FRP reinforcement enhances flexural strength, stiffness, and crack control. However, EBR systems are often limited by premature debonding and environmental exposure. Reviews by Jian et al. [15] and Schober et al. [16] highlight these limitations and suggest that NSM systems offer improved durability and bond reliability. Nevertheless, most existing studies focus on FRP-only systems, with limited attention to hybrid reinforcement strategies.

Prestressed FRP systems further improve structural efficiency by reducing tensile stresses and delaying crack initiation [16,17,18,19]. While effective, these systems involve more complex installation procedures.

### 1.3. Near-Surface-Mounted Reinforcement Techniques

NSM reinforcement has gained attention as an advanced alternative to EBR systems. It involves embedding FRP or steel bars into grooves in the timber surface, resulting in improved bond performance, durability, and fire resistance [20].

Experimental studies [21,22,23,24] have demonstrated significant improvements in flexural capacity, stiffness, and ductility. NSM systems also reduce the likelihood of debonding and provide better protection against environmental effects. Despite these advantages, NSM techniques require precise installation and skilled workmanship. Recent reviews [25] highlight their effectiveness while identifying the need for further research on hybrid systems combining different reinforcement types.

From a polymer chemistry perspective, the development of the FRP–timber bond is governed by the formation of a polymer–wood interphase, which is controlled by adhesive wetting, curing kinetics, and mechanical interlocking. During curing, the epoxy adhesive undergoes an exothermic crosslinking reaction through epoxide ring-opening by amine hardeners, forming a three-dimensional thermoset network. The degree of crosslinking achieved during curing affects the glass transition temperature, stiffness, and viscoelastic behavior of the adhesive, as the formed network structure governs polymer chain mobility and thermomechanical performance, which are critical for transferring shear stresses between the FRP reinforcement and the anisotropic timber substrate [4]. At the molecular interface, polar functional groups in the cured epoxy interact with hydroxyl-containing components of wood, including cellulose, hemicellulose, and lignin, through hydrogen bonding and secondary intermolecular interactions, thereby contributing to interfacial adhesion [5]. Furthermore, the low initial viscosity of the uncured polymer promotes penetration into surface pores and near-surface cellular structures of timber, enhancing mechanical interlocking and the development of an integrated interphase region. Similar polymer infiltration mechanisms, involving in-situ filling of wood cell lumens and interaction with cell wall components, have been reported to improve wood reinforcement and stabilization through polymer–wood interfacial integration [7]. Consequently, the structural performance of FRP-strengthened timber depends not only on the tensile contribution of the FRP reinforcement but also on the polymer structure–property relationship, curing behavior, and interfacial chemistry governing stress transfer and resistance against premature debonding [6].

### 1.4. Numerical Modeling and Analytical Studies

Numerical and analytical studies have significantly contributed to understanding FRP-strengthened timber beams. Research has shown that bond behavior, material variability, and reinforcement configuration strongly influence structural response [26,27,28]. Analytical models [29,30] have improved prediction accuracy, but these are often limited to externally bonded systems. Similarly, studies on shear behavior [31] highlight the importance of combined strengthening approaches. However, existing models lack generalization and rarely address hybrid systems combining NSM reinforcement with FRP sheets.

The above review of literature clearly demonstrates that both NSM and FRP strengthening techniques significantly enhance the performance of timber beams. However, the combined use of NSM FRP bars and FRP sheets remains insufficiently investigated, particularly in terms of flexural strength prediction and interaction mechanisms. Existing models are generally limited to single reinforcement systems and lack generalization across different configurations. Accordingly, this study aims to develop a generalized flexural-strength prediction model for timber beams strengthened with NSM FRP bars and FRP sheets. The specific objectives are to: (i) develop analytical models for reliable prediction of flexural capacity and validate them through experimental testing; (ii) evaluate the effectiveness of hybrid NSM–FRP strengthening systems; and (iii) investigate failure modes and load–deflection behavior. This work contributes to advancing hybrid strengthening strategies and provides a unified framework for the design and analysis of strengthened timber beams.

## 2. Contribution of the Study

This study presents a generalized analytical framework for predicting the flexural strength of timber beams strengthened using a hybrid system comprising NSM FRP bars and externally bonded FRP sheets. Unlike existing models, which are primarily limited to single reinforcement techniques or specific configurations, the proposed model incorporates both embedded and externally bonded reinforcement, capturing their combined effects on stress redistribution and failure mechanisms. The research integrates experimental observations with analytical development to establish a unified prediction approach applicable across different reinforcement ratios, configurations, and material properties. Furthermore, the study provides new insights into hybrid strengthening, demonstrating improved structural efficiency, enhanced ductility, and more reliable performance compared to conventional FRP-only systems. The proposed model is intended to serve as a practical design tool for engineers and researchers, facilitating accurate and efficient assessment of strengthened timber beams and supporting the advancement of modern, sustainable retrofitting strategies.

## 3. Generalized Formulation for Analyzing Strengthened Timber Beams

**Figure 1:**
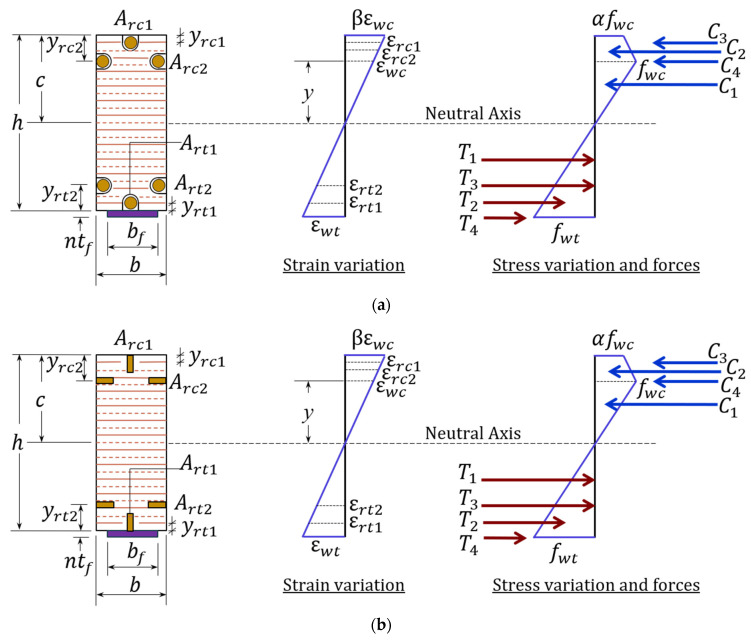
Strain and stress variation and forces in a beam section strengthened against flexure using: (**a**) rebars and (**b**) plates ($C_{1},\;C_{2}=$ compressive forces carried by pre- and post-peak stress–strain regions of the timber, $C_{3},\;C_{4}=$ compressive forces carried by the top and side NSM reinforcements, $T_{1}=$ tensile force carried by the timber, $T_{2},\;T_{3}=$ tensile forces carried by the bottom and side NSM reinforcements, $T_{4}=$ tensile force carried by the FRP, $y_{rc1},\;y_{rc2}$ = distances from the top fiber to the center of the top and side compression NSM reinforcement, $y_{rt1},\;y_{rt2}$ = distances from the bottom fiber to the center of the bottom and side tension NSM reinforcements, c = depth of the neutral axis from the top fiber, y = distance of peak compressive stress from the neutral axis, $\varepsilon_{rc1},\;\varepsilon_{rc2}$ = compressive strains in the top and side NSM reinforcements, $\varepsilon_{rt1},\;\varepsilon_{rt2}$ = tensile strains in the bottom and side NSM reinforcements, $A_{rc1},\;A_{rc2}$ = areas of top and side NSM reinforcements at compression zone, $A_{rt1},\;A_{rt2}$ = areas of bottom and side NSM reinforcements in tension zone, $t_{f}$ = thickness of one FRP layer, and n = number of FRP layers).

**Figure 2:**
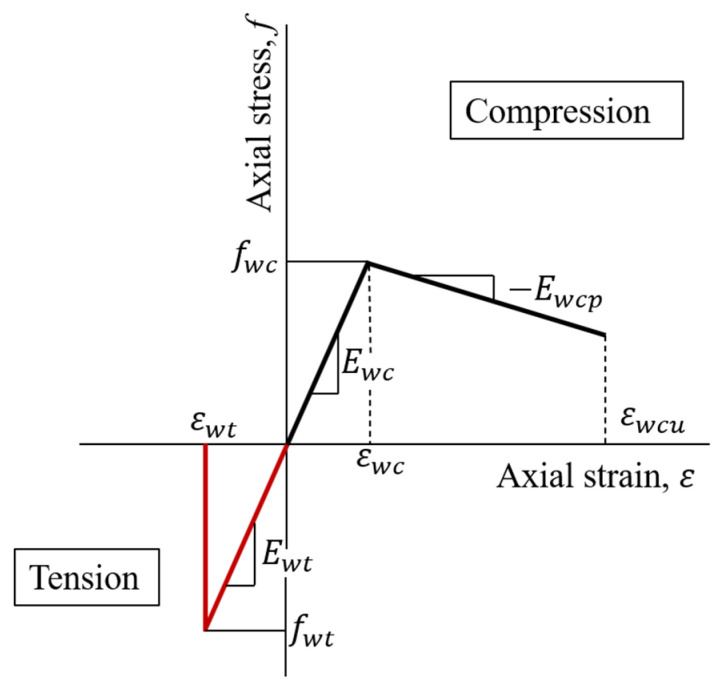
Stress–strain model of timber ($f_{wc}$, $\varepsilon_{wc}$ = ultimate compressive strength of timber and corresponding strain, $f_{wt}$, $\varepsilon_{wt}$ = ultimate tensile strength of timber and corresponding strain, $\varepsilon_{wcu}$ = ultimate compressive strain of timber at failure, $E_{wc}$ = elastic modulus of timber in compression, $E_{wt}$ = elastic modulus of timber in tension, $E_{wcp}$ = post-peak compressive slope).

Material testing was conducted to determine the primary properties of the timber. Considering a timber beam of rectangular section of width b and depth h, strengthened in flexure using NSM bars and FRP sheets attached to the beam’s soffit, as shown in Figure 1. The timber is assumed to follow a linear stress–strain relationship up to the ultimate strengths, $f_{wc}$ and $f_{wt}$ in compression and tension, respectively, with the corresponding strains of $\varepsilon_{wc}$ and $\varepsilon_{wt}$, giving elastic moduli as $E_{wc}$ (${=f}_{wc}/\varepsilon_{wc}$) and $E_{wt}$ (${=f}_{wt}/\varepsilon_{wt}$). Although timber exhibits brittle behavior in tension, leading to sudden failure once its ultimate tensile strength is reached, its compressive stress–strain response beyond the ultimate compressive strength is typically modeled using a linear descending branch with slope $E_{wcp}$ (${=-\gamma E}_{wc}$), as illustrated in Figure 2. This bilinear compression model is consistent with experimental compression test results, and a similar approach was adopted by Malhotra and Bazan [32] based on extensive testing. It is worth noting that this model is straightforward to establish experimentally and remains simple to implement in analysis.

The areas of NSM rebars, used for strengthening, in compression, are $A_{rc1}$ and ${A,}_{rc2}$ and in tension are $A_{rt1}$ and $A_{rt2}$. The suffix 1 in these variables corresponds to the area of NSM rebars provided at the top or bottom faces of the beam, whereas suffix 2 is used for the area of NSM rebars provided on the side faces of the beam. Irrespective of the material (steel or FRP), the rebars are assumed to be linearly elastic with elastic modulus of $E_{r}$ and strength $f_{rf}$, which is the yield strength for steel bars and ultimate strength corresponding to fracture strain ($\varepsilon_{rf}$) for the FRP bars. However, steel bars yield after crossing the yield strain ($\varepsilon_{rf}$). Although only one NSM compression bar is shown in Figure 1, more than one bar can be provided depending on the width of the beam. The FRP sheets, of $E_{f}$ elastic modulus, used for strengthening the beam are $b_{f}$ wide. The layer thickness of the FRP sheet is $t_{f}$, and the beam is strengthened using n layers. It is worth noting that the timber beam is also strengthened in shear to prevent premature shear failure. When the flexural capacity is increased beyond the inherent shear strength, shear reinforcement becomes essential; otherwise, the beam may fail by mid-depth splitting caused by the timber’s limited longitudinal shear resistance.

For the sectional analysis of the beam, the compressive and tensile force components in the timber, NSM reinforcement, and FRP sheet, as well as their respective distances from the top fiber, were calculated and are presented in Table 1 through Equations (1)–(8).

$C_{1}$, $C_{2}$, $C_{3}$, and $C_{4}$ represent the compressive forces carried by the timber lower compression zone (pre-peak stress–strain region), timber upper compression zone (post-peak stress–strain region), top NSM reinforcement, and side NSM reinforcement, respectively, whereas $T_{1}$, $T_{2}$, $T_{3}$, and $T_{4}$ represent the tensile forces carried by the timber, FRP, bottom NSM reinforcement, and side NSM reinforcement, respectively, $y_{c1}-y_{c4}$ = distances from top fiber to $C_{1}-C_{4}$, $y_{t1}-y_{t4}$ = distances from top fiber to $T_{1}-T_{4},$ b = width of the rectangular timber beam cross-section, h = depth (overall height) of the rectangular timber beam cross-section, $f_{wc}$ = ultimate compressive strength of timber, $f_{wt}$ = ultimate tensile strength of timber, $\varepsilon_{wc}$ = compressive strain of timber at $f_{wc}$, $\varepsilon_{wt}$ = ultimate tensile strain of timber, $E_{wc}$ = elastic modulus of timber in compression (=$f_{wc}/\varepsilon_{wc}$), $E_{wt}$ = elastic modulus of timber in tension (=$f_{wt}/\varepsilon_{wt}$), $E_{wcp}$ = post-peak compressive slope (=−$\gamma E_{wc}$), γ = reduction factor for post-peak slope, $A_{rc1}$ = area of NSM reinforcement at compression face, $A_{rc2}$ = area of NSM reinforcement at side faces in compression zone, $A_{rt1}$ = area of NSM reinforcement at tension face, $A_{rt2}$ = area of NSM reinforcement at side faces in tension zone, $E_{r}$ = elastic modulus of reinforcement, $f_{rf}$ = yield strength (steel) or ultimate strength (FRP), $\varepsilon_{rf}$ = yield strain (steel) or fracture strain (FRP), $E_{f}$ = elastic modulus of FRP sheet, $b_{f}$ = width of FRP sheet, $t_{f}$ = thickness of one FRP layer, and n = number of FRP layers.

(9) α=1−γ(β−1)

(10) $$\gamma =\frac{E_{wcp}}{E_{wc}}=\frac{1}{r}$$

(11) $$y=\frac{c}{\beta }$$

(12a) $$\rho_{rc1}=\frac{A_{rc1}}{bh}$$

(12b) $$\rho_{rc2}=\frac{A_{rc2}}{bh}$$

(13a) $$\rho_{rt1}=\frac{A_{rt1}}{bh}$$

(13b) $$\rho_{rt2}=\frac{A_{rt2}}{bh}$$

(14a) $$\varepsilon_{rc1}=\beta \varepsilon_{wc}\left(1-\frac{y_{rc1}}{c}\right)\leq \varepsilon_{rf}$$

(14b) $$\varepsilon_{rc2}=\beta \varepsilon_{wc}\left(1-\frac{y_{rc2}}{c}\right)\leq \varepsilon_{rf}$$

(15a) $$\varepsilon_{rt1}=\beta \varepsilon_{wc}\left(\frac{h}{c}-1-\frac{y_{rt1}}{c}\right)\leq \varepsilon_{rf}$$

(15b) $$\varepsilon_{rt2}=\beta \varepsilon_{wc}\left(\frac{h}{c}-1-\frac{y_{rt2}}{c}\right)\leq \varepsilon_{rf}$$

(16) $$A_{f}=nt_{f}b_{f}$$

(17) $$c=\frac{h}{1+\frac{\varepsilon_{wt}}{{\beta \varepsilon }_{wc}}}$$

For equilibrium, equating the sum of forces to zero:

(18) $$\sum_{i=1}^{4}C_{i}-\sum_{i=1}^{4}T_{i}=0$$

Putting the values of the compressive and tensile forces from Equations (1a) through (8a), gives:

(19) A+B=0

where

(20) $$A=\frac{1}{2}\beta^{2}-\left(1+r\right)\beta +\frac{1}{2}\left(1+r+r\frac{\varepsilon_{wt}}{\varepsilon_{wc}}\frac{f_{wt}}{f_{wc}}\right)$$

(21) $$B=r\beta \frac{E_{r}}{f_{wc}}\left(1+\frac{\varepsilon_{wt}}{\beta \varepsilon_{wc}}\right)\left(\varepsilon_{rt1}\rho_{rt1}+\varepsilon_{rt2}\rho_{rt2}-\varepsilon_{rc1}\rho_{rc1}-\varepsilon_{rc2}\rho_{rc2}\right)+\varepsilon_{wt}\frac{{rE}_{f}A_{f}}{f_{wc}bc}\beta$$

Equation (18) is separated into two components, A and B, where A represents the contribution of the timber beam and B corresponds to the strengthening materials, including the NSM reinforcement and FRP sheets. This equation can be iterated to find the value of β, thus giving the depth of the neutral axis, c. However, for control beam (i.e., unstrengthened beam), B=0, thus the value of β can be determined from A=0, which is a quadratic equation in β, thus giving:

(22) $$\beta =\left(1+r\right)-\sqrt{r^{2}+r-r\frac{\varepsilon_{wt}}{\varepsilon_{wc}}\frac{f_{wt}}{f_{wc}}}\geq 1$$

If $E_{wc}=E_{wt}$ (which is most common) and $f_{wc}=f_{wt}$ then for the control beam, β=1. Additionally, the timber strength should be such that $f_{wt}\geq f_{wc}$.

The ultimate moment of resistance of the strengthened timber beam, $M_{us}$, can be calculated using:

(23) $$M_{us}=\sum_{i=1}^{4}C_{i}y_{ci}-\sum_{i=1}^{4}T_{i}y_{ti}$$

For the control beam, the ultimate moment of resistance, $M_{uc}$, can be alternatively calculated using:

(24) $$M_{uc}=\frac{2}{3}C_{1}\left(h-c+y\right)+\frac{1}{3}C_{2}\left[c+2h-\frac{\left(\alpha +2\right)}{\left(\alpha +1\right)}\left(c-y\right)\right]$$

The adhesive in the FRP–timber system serves two functions: it acts as the polymer matrix that maintains fiber integrity and as the bonding medium that transfers stresses between the FRP reinforcement and timber substrate. Due to the thin adhesive layer relative to the timber section and the FRP thickness, and the limited penetration depth into the wood structure, its direct contribution to the global bending resistance is not considered separately. Instead, the adhesive contribution is reflected through the effectiveness of the FRP–timber interface and the associated stress-transfer mechanism. A detailed investigation of adhesive properties and interface mechanics is an important area for future research to further clarify their influence on the performance of polymer-based timber-strengthening systems.

## 4. Materials and Their Properties Used for Strengthening

The materials utilized in this study’s experimental work included structural timber, GFRP sheets, NSM GFRP bars, and epoxy adhesives.

### 4.1. Timber

Material testing was conducted to determine the fundamental properties of the timber, including density, moisture content, and compressive and tensile strengths, as described in the following sections. The tests were performed in accordance with ASTM D4442-20 [33] for moisture content, ASTM D2395-17 [34] for density, and ASTM D143-25 [35] for the determination of compressive and tensile strengths.

#### 4.1.1. Density and Moisture Content

Four samples were extracted from each specimen to evaluate moisture content, density, and compressive strength. The mean density was recorded as 515 ± 9 kg/m^3^. The average moisture content was measured at 8.0% ± 0.11%.

#### 4.1.2. Compression Tests

**Figure 3:**
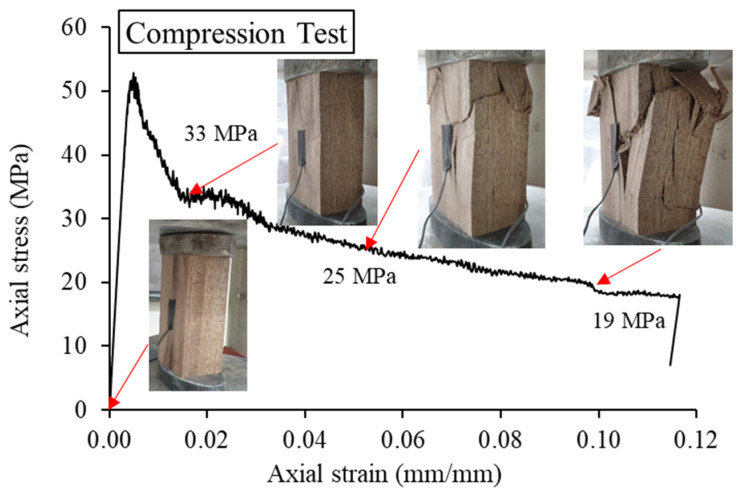
Stress–strain variation of timber in compression with damage progression in test specimens.

Several timber specimens measuring 52 mm × 76 mm × 142 mm (height) were tested under axial compression, with the grains oriented parallel to the applied compressive force. Although the specimen size differs slightly from those adopted in previous studies, this geometry was selected based on material availability. Representative stress–strain response of one of the specimens is presented in Figure 3, illustrating the typical compressive behavior of timber. The corresponding damage states at selected load (stress) levels, where noticeable changes occur, are also indicated in the figure. The stress–strain response remains nearly linear up to approximately 85–90% of the peak stress. No visible external damage was observed up to the peak load; however, internal splitting was detected by audible cracking, with only minor surface indications. Beyond the peak stress, a sudden and steep reduction in load, on the order of 35–50%, was observed at compressive strains of approximately 1–2%. This drop is attributed to the growth of splitting along the longitudinal grain direction. Following this initial drop, a partial recovery in load (and hence stress) occurred, after which the stress gradually decreased with increasing strain up to a strain level exceeding 10%. During this phase, the material exhibited a slow post-peak softening response, accompanied by progressive splitting and fracture, indicating considerable ductility, which is an inherent characteristic of timber under compression parallel to the grain. The final failure occurred due to compression and shearing. For the purpose of constitutive modeling of timber in compression, only the stress–strain response up to the first post-peak load drop should be considered, as the subsequent load recovery and extended softening phase are governed by progressive fracture mechanisms rather than stable material behavior.

#### 4.1.3. Tension Tests for Tensile Strength Along the Fiber Direction

Dog-bone-shaped coupons were employed to determine the tensile strength of structural timber parallel to the grain. This geometry was selected to promote a uniform stress distribution within the gauge section and to ensure that failure occurs away from the grips. The coupons were machined such that the wood fibers were oriented along the longitudinal axis, with a reduced central section to localize tensile failure within the test zone. Unlike metallic materials, for which standardized dog-bone geometries and gripping systems are well established, the direct tension testing of timber presents significant challenges. Owing to the relatively low compressive strength and anisotropic nature of wood, inappropriate specimen proportions or gripping arrangements may induce premature failure in the grip region, either through local crushing or splitting, rather than tensile fracture in the gauge section. Consequently, the selection of coupon geometry, specifically the proportions of the grip and neck regions, and the gripping system required careful optimization.

**Figure 4:**
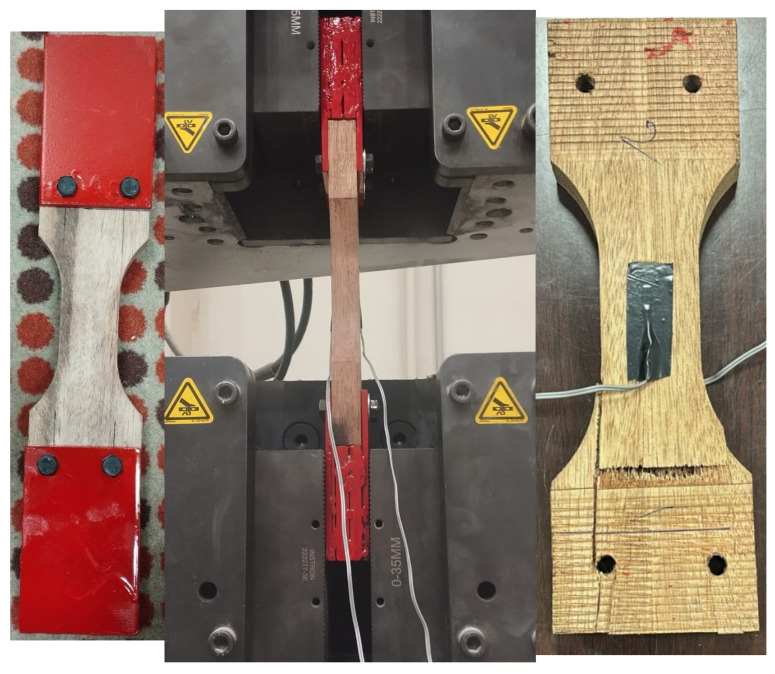
Some of the failed dog-bone specimens in the tension test.

**Figure 5:**
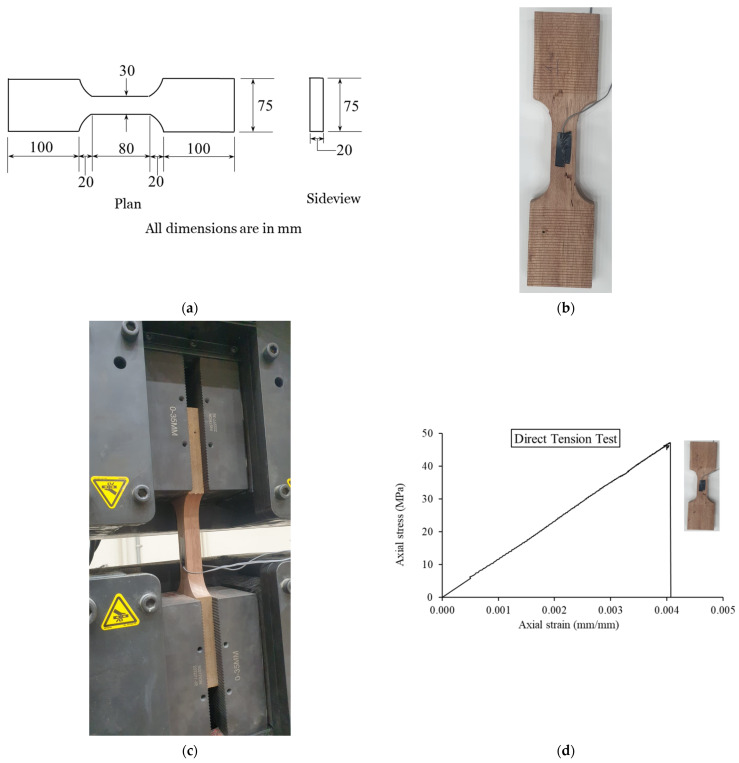
Tension test for the tensile strength of the timber: (**a**) coupon dimensions; (**b**) coupon after test with strain gage attached; (**c**) test setup; and (**d**) stress–strain plot.

Initial trials employed mechanically fastened grips using two bolts of diameter 8 mm in each grip, with a neck section of 40 mm (width) × 20 mm (thickness). These specimens failed prematurely due to the splitting of timber around the bolt holes, as the tensile load was transferred through localized bearing stresses. Subsequent tests using direct machine grips on specimens of the same geometry resulted in compressive failure at the grip region, preventing the development of uniform tensile stress in the gauge section. Representative specimens and failure modes from these trials are shown in Figure 4. Following several iterations, the coupon geometry was modified by reducing the neck (test) section and increasing the grip area, as shown in Figure 5. This configuration was found to be adequate for achieving tensile failure within the gauge length. The final specimen was 320 mm long, with a gauge length of 80 mm and a uniform width and thickness of 30 mm and 20 mm, respectively. The grip regions were 100 mm long and 75 mm wide. It should be noted that these dimensions are specific to the timber species and grade tested in this study and may require adjustment for other timber types. Although a very small neck section is theoretically desirable for stress localization, excessively reducing the cross-sectional area increases sensitivity to load eccentricity and alignment imperfections, making it difficult to ensure uniform uniaxial tension. Therefore, balanced geometry was adopted to ensure both reliable gripping and stable tensile loading. Alternatively, the tension can be performed using test specimens that can be pulled without gripping but this requires a special test setup [36].

The specimens were tested in a universal testing machine (Instron, Norwood, MA, USA) under displacement-controlled loading, with tension applied parallel to the grain until failure. Strain in the gauge section was measured using an electrical resistance strain gauge bonded at the mid-length of the neck (Figure 4 and Figure 5). A representative stress–strain response is presented in Figure 5d, showing an almost linear relationship up to failure. All specimens failed by tensile fracture within the gauge section, confirming the adequacy of the adopted specimen geometry and gripping system.

#### 4.1.4. Constitutive Modeling

A wide range of constitutive models has been proposed in the literature to represent the compressive stress–strain behavior of timber parallel to the grain, ranging from the simplest elastic–perfectly plastic (bilinear) idealization [37] to more advanced nonlinear formulations incorporating gradual yielding and damage evolution [32,38]. The selection of an appropriate model largely depends on the intended level of accuracy and the application context, particularly whether pre-peak or post-peak behavior is of interest.

**Figure 6:**
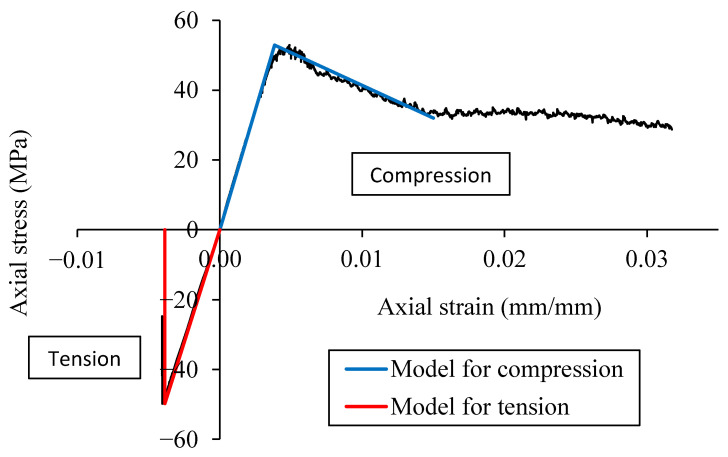
Comparison between the proposed constitutive models and representative experimental stress–strain curves (along the fiber direction) obtained in this study.

The experimental results of the present study indicate that a bilinear stress–strain model with a descending second branch provides a close representation of the observed compressive response up to the onset of unstable damage (Figure 6). A similar modeling approach was previously adopted by Malhotra and Bazan [32] for timber under compression parallel to the grain. In the proposed model, the first branch represents the linear elastic response up to the peak stress, while the second branch captures the post-peak softening associated with longitudinal grain splitting. Based on the test results, the magnitude of the negative slope of the second branch varies widely as a function of the initial elastic stiffness ratio, reflecting variability in material heterogeneity and fracture progression.

In contrast, the tensile behavior of timber parallel to the grain exhibits an essentially linear elastic response up to sudden brittle failure, as shown in Figure 5d. No measurable plasticity or stable damage evolution was observed prior to fracture. The tensile modulus is nearly identical to the elastic modulus obtained from compression tests, indicating comparable stiffness in both loading regimes. Consequently, the tensile response is appropriately modeled using a linear elastic law with a sudden stress drop at failure.

Figure 6 compares the proposed constitutive models with representative experimental stress–strain curves obtained in this study, demonstrating good agreement in both compression and tension. The adopted modeling framework provides a rational balance between simplicity and accuracy, making it suitable for analytical and numerical simulations where stable pre-peak behavior and the onset of damage are of primary interest.

The compressive stress–strain behavior of timber parallel to the grain is represented using a bilinear model with post-peak softening (Figure 2), defined as:

(25) $$f=\{\begin{array}{c} 0\;\mathrm{for}\;\varepsilon \leq \varepsilon_{wt}\\ \varepsilon E_{wt}\;\mathrm{for}\;\varepsilon_{wt}\leq \varepsilon \leq 0\\ \varepsilon E_{wc}\;\mathrm{for}\;0\leq \varepsilon \leq \varepsilon_{wc}\\ f_{wc}+\left(\varepsilon -\varepsilon_{wc}\right)E_{wcp}\;\mathrm{for}\;\varepsilon_{wc}\leq \varepsilon \leq \varepsilon_{wcu} \end{array}$$

where f is the compressive stress parallel to grain, ε is the strain in timber, other variables are defined above in Figure 2. Based on the experimental results of this study, the softening modulus in compression $E_{wcp}$ may be expressed as a fraction of the initial elastic modulus of timber in compression (i.e., $E_{wcp}=\alpha E_{wc}$) considering specimen-to-specimen variability and the extent of longitudinal grain splitting. Only the response up to $\varepsilon_{wcu}$ (Figure 2) is considered in the constitutive model for compression. Beyond this point, the material behavior is governed by unstable fracture and progressive splitting, and is therefore excluded from modeling. The elastic modulus in tension $E_{wt}$ was found to be nearly identical to that obtained from compression tests (Figure 6), justifying the use of a common modulus $E_{wc}$ for both loading regimes.

### 4.2. GFRP Sheets

GFRP sheets provide an efficient and cost-effective solution for strengthening timber beams by enhancing both flexural and shear capacity while maintaining a high strength-to-weight ratio. They are inherently corrosion-resistant, durable, and moisture-resistant, making them well suited for application in aggressive or variable environmental conditions. Their relatively low density and flexibility facilitate ease of installation with minimal surface preparation and without significant alteration to the original member geometry, thereby preserving the architectural integrity of timber structures.

From a mechanical standpoint, GFRP exhibits a modulus of elasticity and tensile strength that are more compatible with timber compared to stiffer alternatives such as CFRP. This improved compatibility promotes a more uniform stress distribution and reduces stress concentrations at the timber–FRP interface, which can otherwise lead to premature debonding or localized damage. In contrast, the very high stiffness and strength of CFRP may result in inefficient material utilization and a brittle, over-reinforced response when applied to timber, particularly when failure is governed by the relatively weak tensile or perpendicular-to-grain properties of wood. Furthermore, GFRP demonstrates good fatigue resistance and contributes to reduced maintenance requirements over the service life of the structure. It can be effectively combined with other strengthening techniques, such as NSM reinforcement or transverse confinement systems, to further enhance structural performance. With appropriate protective coatings, its fire resistance can also be improved. Overall, GFRP represents a versatile, mechanically compatible, and sustainable solution for strengthening solid timber beams, which are used in the present study. In this study, GFRP laminates of 1.3 mm thickness were used, having a tensile strength of 422 MPa, a modulus of elasticity of 25 GPa, and a fracture strain of 1.69%.

### 4.3. NSM Bars

Near-surface-mounted FRP bars provide an efficient and reliable strengthening technique for timber beams by enhancing bond performance and load transfer through embedment in pre-cut grooves filled with adhesive. This configuration ensures effective stress transfer between the reinforcement and the timber substrate, resulting in improved flexural capacity and, in some cases, enhanced resistance to shear-induced splitting. Compared to externally bonded reinforcement, NSM systems are less susceptible to premature debonding and are inherently better protected against environmental exposure, mechanical damage, and elevated temperatures due to their embedment within the timber section. The internal placement of NSM bars promotes a more uniform stress distribution and improved anchorage, thereby delaying crack initiation and propagation. In addition, the method preserves the member’s external surface, maintaining its structural and architectural integrity. From a practical standpoint, NSM reinforcement is relatively easy to install, requires smaller quantities of adhesive than externally bonded systems, and allows efficient use of FRP materials, making it a cost-effective and durable strengthening solution.

With regard to material selection, GFRP bars were preferred over CFRP in the present study. Although CFRP offers significantly higher stiffness and tensile strength, its use in timber strengthening may lead to incompatibility in stiffness, resulting in stress concentrations at the timber–FRP interface and premature failure of the timber substrate or bond line before full utilization of the reinforcement. In contrast, GFRP exhibits a modulus of elasticity that is closer to that of timber, enabling more compatible deformation behavior and improved stress sharing between materials. This compatibility is particularly important in timber members, where failure is often governed by tensile rupture parallel to grain or splitting perpendicular to grain. Consequently, GFRP provides a more balanced and efficient reinforcement strategy for timber beams. In this study, NSM GFRP bars with a diameter of 14 mm were used, having a tensile strength of 680 MPa, a modulus of elasticity of 48 GPa, and a fracture strain of 1.42%.

### 4.4. Epoxy Adhesives

Epoxy adhesives are crucial for strengthening timber beams with FRP sheets and NSM bars, providing strong bonding, excellent load transfer, and gap-filling capabilities. They ensure high interfacial adhesion, preventing premature debonding and enhancing the composite action between the timber and reinforcement. Epoxies exhibit superior mechanical strength, durability, and resistance to moisture, chemicals, and temperature variations, making them ideal for long-term structural performance. Their thixotropic nature allows easy application in grooves for NSM bars, ensuring proper embedding, while their flexibility and shrinkage resistance improve adhesion with FRP sheets. Additionally, epoxy adhesives reduce stress concentrations and enable efficient force distribution, enhancing timber beams’ overall strength, stiffness, and service life.

## 5. Experimental Validation

Timber beams are widely used in structural applications, and their behavior under applied loads is crucial for ensuring safety and performance. To assess their structural response, experimental testing was conducted using reduced-scale specimens subjected to controlled loading conditions. These tests involved applying gradually increasing loads to simulate real-world forces, allowing the observation of failure modes, crack propagation, and deformation characteristics.

The strengthening technique adopted is a combination of NSM FRP rebars and externally bonded FRP sheets, which were incorporated to enhance the beams’ load-carrying capacity and ductility. The results from these tests provide valuable insights into the mechanical properties of timber and the effectiveness of reinforcement strategies, aiding in the development of improved design guidelines and strengthening methods.

Specimens were tested primarily to establish behavioral trends and to validate the developed strengthening model for timber beams; therefore, replicate tests were not conducted.

### 5.1. Flexural Strengthening

**Figure 7:**
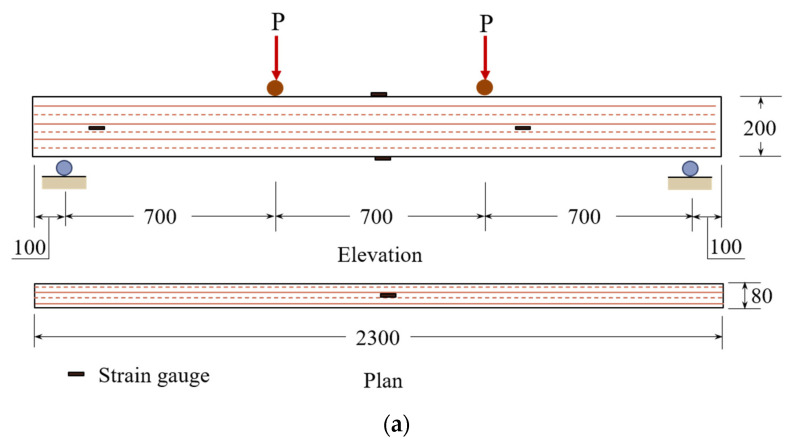

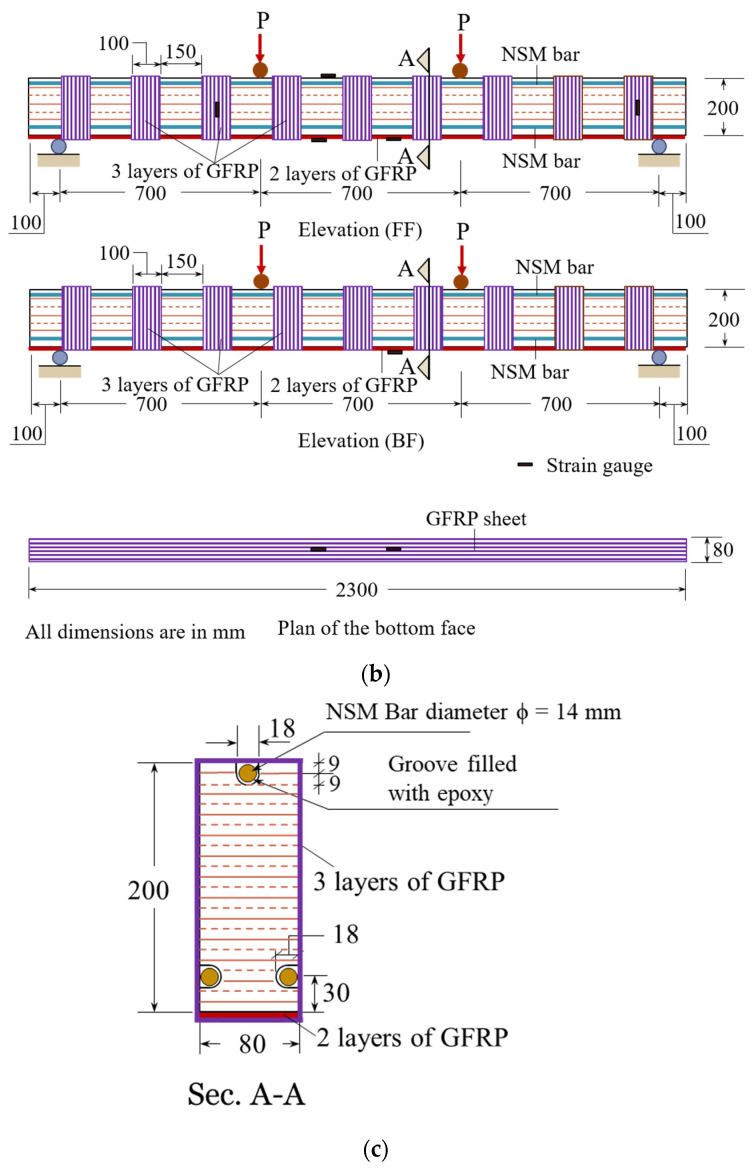
Details of timber beams tested for flexure: (**a**) control beam and (**b**,**c**) strengthened beam (unit: mm).

The size of the control beam specimens for flexural strengthening was 80 × 200 × 2300 mm, as shown in Figure 7a. The control beams were strengthened using 14 mm diameter GFRP NSM bars and GFRP sheets. For flexural strengthening of beams, one NSM bar was inserted in a groove of 18 mm square in the compression zone (i.e., at the top), and two NSM bars were inserted in the grooves of the same size on the sides at 30 mm above the bottom face of the beams in the tension region, as shown in Figure 7b,c. The grooves with NSM bars were filled with adhesive. Subsequently, GFRP sheets were bonded to timber beams after careful surface preparation, adhesive selection, and proper application techniques. The timber surface was first cleaned to remove dust, dirt, and loose particles, followed by sanding to enhance roughness and improve adhesion. It was ensured that the timber had a moisture content below 12–15%, as excessive moisture can weaken the bond. Once sanded, all dust was removed using compressed air. The epoxy resin was employed for bonding GFRP to the timber surface. Before application, GFRP sheets were cut to the required dimensions (80 mm wide), and a uniform layer of epoxy was applied to both the timber surface and the GFRP sheet, ensuring complete saturation of the fabric. Once the adhesive was applied, the GFRP sheet was carefully positioned on the prepared surface while the epoxy remained wet. A roller (squeegee) was used to press the sheet, ensuring firm contact and removing any air bubbles. If needed, additional epoxy was applied to ensure complete wetting. The procedure was repeated for the application of the second layer of GFRP sheet. Uniform pressure was applied using weights until the adhesive cured. Curing was performed for 72 h at room temperature, during which the bonded area remained undisturbed. Thus, two layers of GFRP sheets were applied to the tension face (bottom) of the beam. Subsequently, three layers of 100 mm wide vertical closed GFRP strips were externally bonded at a center-to-center spacing of 250 mm, following the same procedure. These strips were provided to anchor the bottom FRP sheet, restrain splitting, and enhance resistance against longitudinal splitting.

The strain gauges were strategically placed to capture strain variations along the beam’s length and depth. The bottom fiber (tension zone) at mid-span is a critical location where strain gauges were attached to measure tensile strain, as this is where maximum bending occurs. Additionally, strain gauges were placed directly on the GFRP sheet at mid-span to monitor strain transfer between the timber and reinforcement. To verify strain compatibility, an additional strain gauge was installed near the neutral axis at mid-depth to confirm that the neutral axis remained at the expected location. All NSM bars had a strain gage at their midspan. The locations of various strain gauges are shown in Figure 7.

**Figure 8:**
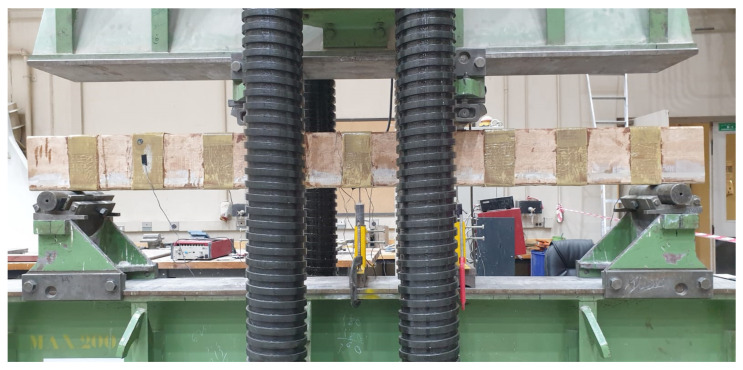
Test setup showing flexure-strengthened timber beam during test.

The control and strengthened timber beams were tested under simply supported conditions using steel roller supports. Each beam had a clear span of 2100 mm with 100 mm overhangs at both ends (Figure 7). The beams were subjected to four-point bending through two concentrated loads applied via steel rollers, spaced 700 mm apart, thereby creating a constant-moment region between the loading points. The load was applied under displacement control using an Amsler testing machine (Alfred J. AMSLER & Co., Schaffhouse, Switzerland) at a rate of 1 mm/min. Vertical deflections were measured using two LVDTs positioned symmetrically about the midspan, as shown in Figure 8, to capture the global flexural response of the beam. Load, deflection, and strain data were recorded continuously at 0.5 Hz using a data acquisition system. The test setup is shown in Figure 8.

#### 5.1.1. Control Beam

**Figure 9:**
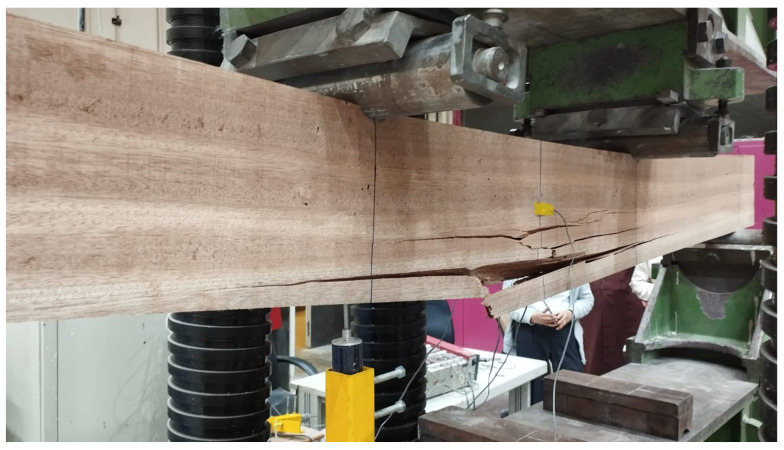
Failure of the flexure control beam.

The control (unstrengthened) timber beam failed in a classical flexure-dominated mode characterized by tensile rupture parallel to the grain. Cracking initiated at the extreme tension fiber in the constant-moment region, where the maximum bending tensile stress occurred, and propagated rapidly upward along the grain direction (Figure 9). The crack development was sudden and unstable, reflecting the inherently brittle nature of timber in tension parallel to grain and its limited post-peak deformation capacity. Following crack initiation, the load-carrying capacity dropped abruptly with minimal warning, confirming that failure was governed by the tensile strength of timber. No significant diagonal shear cracking was observed, indicating that the shear stress remained below the longitudinal splitting threshold at ultimate load. Localized indentation beneath the loading rollers was also observed, attributable to compressive stresses perpendicular to the grain. This bearing deformation is typical in timber beams due to the relatively low compressive strength perpendicular to the grain; however, it did not govern the global failure mechanism.

This tensile rupture mode is widely recognized as the predominant flexural failure mechanism for solid timber beams subjected to bending and has been consistently reported in previous experimental investigations, e.g., Forest Products Laboratory [39]. However, solid timber beams may also fail by compressive crushing in the extreme compression zone when compressive stress exceeds the crushing strength parallel to grain, often leading to progressive plastic-like deformation and localized fiber buckling prior to ultimate failure [39,40]. Depending on the relative tensile and compressive capacities, bending failure may occur either as a sudden brittle fracture on the tension side or as a more gradual compression-controlled response. Additional flexural failure mechanisms include bearing or local crushing at load or support points that can interact with bending stresses [40,41]. Consequently, the governing flexural failure mode in solid timber beams depends on the balance among tensile rupture strength, compressive crushing resistance, and local stress concentrations.

**Figure 10:**
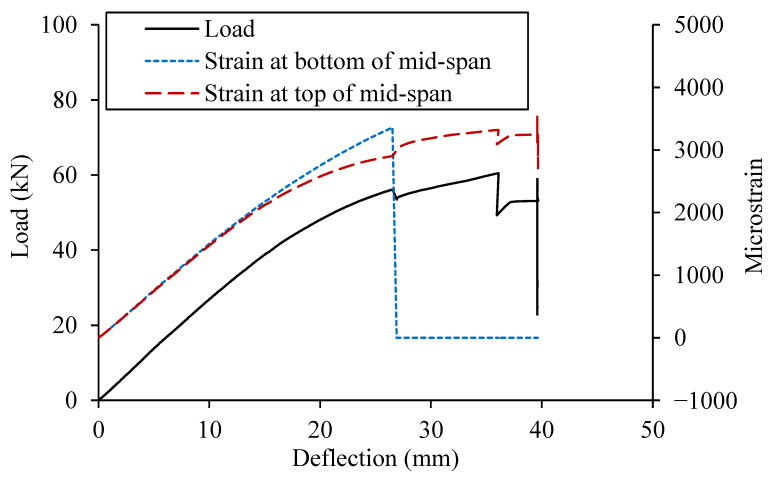
Load-deflection and strain plots of the flexure control beam.

Figure 10 presents the variation in load and corresponding strains at the top (compression zone) and bottom (tension zone) of the control beam tested in flexure. For clarity in plotting, the compressive strain at the top fiber, originally negative, is shown as positive after sign conversion. The load–deflection response is initially linear up to approximately 50% of the ultimate load, beyond which nonlinear behavior becomes evident. At a deflection of 26.9 mm, a slight drop in load occurs at 56.1 kN (about 93% of the peak load), indicating the onset of damage. However, the load increases further with increasing deflection, reaching a peak of 60.4 kN (bending moment = 21.2 kN·m) at 36.0 mm deflection. Up to this stage, the compressive strain at the top fiber follows a trend consistent with the applied load.

In the initial linear range, the tensile strain at the bottom fiber closely matches the compressive strain at the top, indicating symmetric strain distribution about the neutral axis and confirming that the modulus of elasticity in tension and compression is essentially the same within the elastic regime. As loading progresses beyond the linear range, the tensile strain begins to deviate and increases at a higher rate than the compressive strain. This divergence indicates a shift of the neutral axis toward the compression zone, caused by stiffness degradation in the tension region due to microcracking and progressive timber damage. At a deflection of 26.9 mm, a sudden drop in tensile strain is observed at approximately 3360 microstrain, corresponding to tensile fracture of the timber at the bottom fiber and failure of the strain gauge. Following this, the compressive strain continues to increase in line with the load until final failure. The maximum compressive strain at failure was approximately 3535 microstrain at a deflection of 39.6 mm. This behavior confirms that failure was governed by tensile rupture in the bottom fiber, followed by continued load resistance through the compression zone, with a progressive neutral axis shift reflecting stiffness degradation in the tension region.

The predicted moment of resistance calculated using Equation (24) is 20.7 kN·m, corresponding to a load capacity of 59.0 kN, which is 2% lower than the experimental value of 60.4 kN.

#### 5.1.2. Strengthened Beam

**Figure 11:**
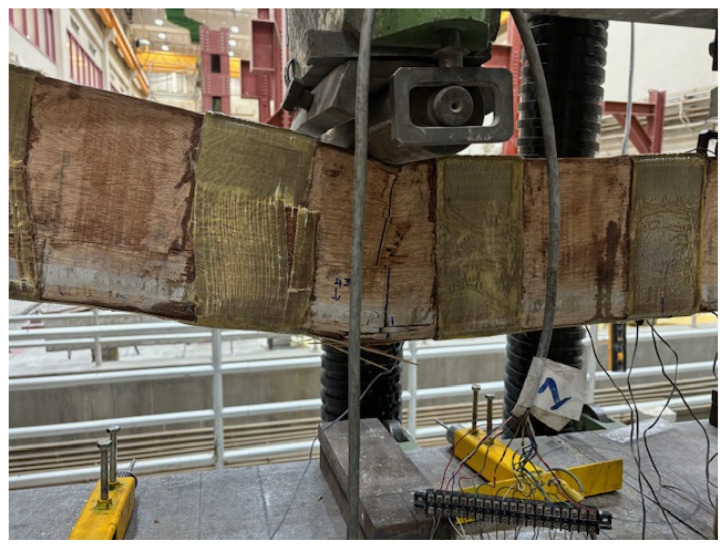

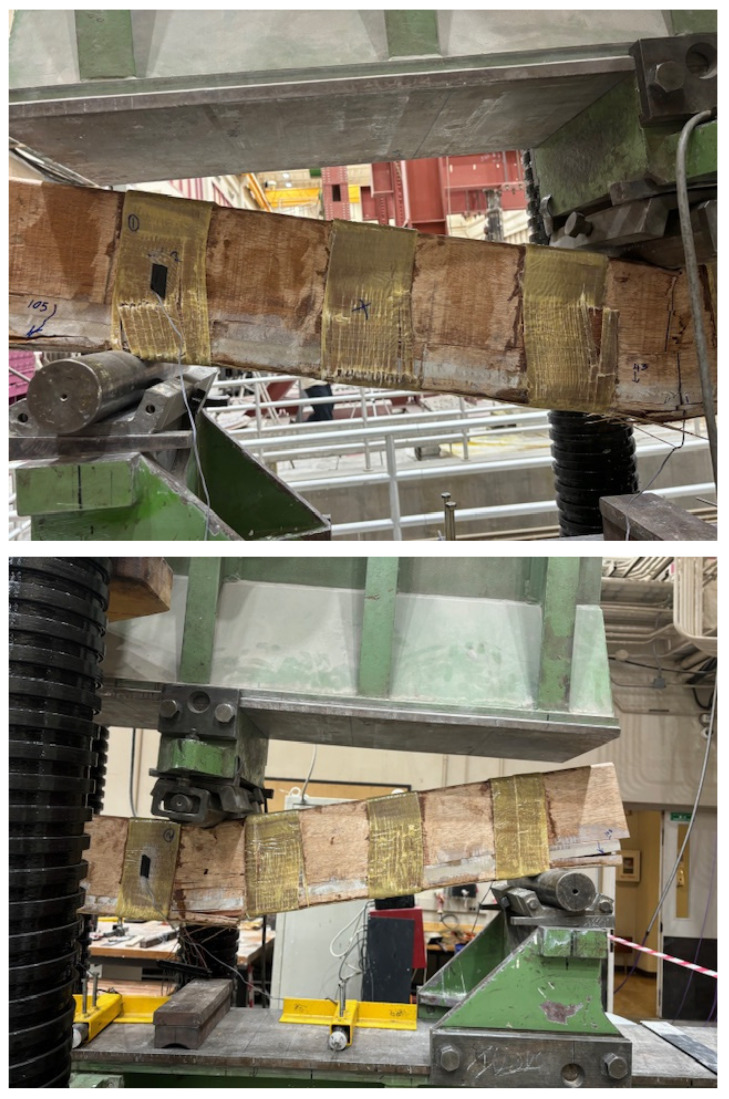
Failure of the flexure-strengthened beam.

The strengthened beam exhibited predominantly flexure-governed failure initiated by tensile rupture of timber at the extreme bottom fiber in the constant-moment region (Figure 11). Despite the presence of two externally bonded (EB) GFRP sheets at the soffit, cracking first occurred in the timber substrate, indicating that the tensile capacity of wood governed prior to full utilization of the composite reinforcement. The initiation of bottom fiber rupture triggered localized intermediate crack (IC) debonding of the soffit GFRP sheets, attributable to stress concentration and loss of composite action following substrate cracking. With further increase in load, the tensile cracks propagated vertically and extended into the shear span, leading to a redistribution of internal stresses. As longitudinal shear stresses intensified, longitudinal splitting developed parallel to the grain within the shear zone. The three-layer vertical closed GFRP strips, intended to restrain splitting and improve shear confinement, fractured progressively as the splitting crack widened. This indicates that the shear strengthening system was effectively engaged but ultimately reached its tensile capacity under the induced splitting stresses.

At higher load levels, localized bearing damage was observed beneath the loading rollers, as indentation of the timber surface. This localized crushing reflects the relatively low compressive strength perpendicular to the grain and contributes to stress redistribution, but is not the primary cause of global failure. In the overhang region, pronounced longitudinal splitting occurred, evidenced by relative slip along the upper face of the longitudinal groove accommodating the bottom NSM GFRP bars. The timber strip located between the soffit and the bottom face of the NSM groove progressively detached and rotated outward, indicating a loss of shear transfer across the weakened section. This behavior confirms that splitting was governed by longitudinal shear stresses interacting with the geometric discontinuity introduced by the NSM slot.

Notably, no rupture of the two side NSM GFRP tension bars was observed, consistent with their substantially higher tensile strength relative to the timber substrate. Their integrity at failure suggests that the governing mechanism remained timber-controlled rather than FRP rupture-controlled. Similarly, the single top NSM GFRP bar remained intact, confirming that compressive zone stresses and bond demands in that region did not exceed its capacity. Debonding of the externally bonded GFRP sheets was limited to intermediate crack (IC) debonding within the pure bending zone, while bond integrity was maintained along the remaining beam length, particularly in the shear spans. This behavior indicates effective anchorage provided by the vertical GFRP U-wraps and efficient shear transfer outside the primary flexural cracking region.

Overall, the failure sequence demonstrates that the applied strengthening configuration significantly enhanced flexural capacity and delayed splitting; however, ultimate failure was still governed by tensile rupture of the timber followed by shear-induced longitudinal splitting, rather than FRP rupture. The response highlights the critical roles of timber tensile strength and splitting resistance in determining the ultimate capacity of solid timber beams strengthened with a hybrid system of externally bonded FRP and NSM GFRP bars.

**Figure 12:**
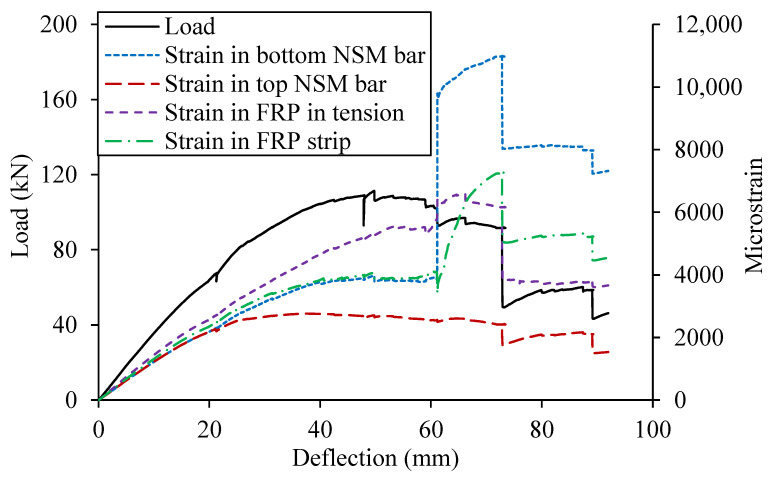
Load-deflection and strain plots of flexure-strengthened beam.

Figure 12 presents the variation of load with midspan vertical deflection, along with the corresponding strains in the bottom and top NSM bars, the externally bonded FRP in the tension zone, and the vertical FRP strip for which the recorded strain was the highest. For consistency, compressive strains are plotted as positive after sign conversion. The load-deflection response is initially linear up to approximately 50% of the ultimate load, beyond which nonlinear behavior is observed. The strain response in all components also remains linear within this elastic range. Beyond the elastic limit, the strains in the bottom NSM bars and the tension-side FRP continue to follow a trend similar to the applied load, indicating effective stress transfer and composite action. The strain in the top NSM bar remains comparatively low throughout the loading, consistent with its location in the compression zone.

The tensile strain in the externally bonded FRP is higher than that in the bottom NSM bars, as the FRP is located at the extreme tension fiber, whereas the NSM bars are embedded approximately 30 mm above the bottom surface. This difference reflects the strain gradient across the depth of the section. The beam achieved a peak load of 111.4 kN (84% higher than the control beam). At a deflection of 61.1 mm, a slight drop in load is observed, corresponding to the initiation of tensile fracture in the timber at the bottom fiber. The onset of longitudinal splitting leads to localized separation of timber, which in turn induces a sudden increase in tensile strain in the FRP (as it is pushed outward by split timber) due to redistribution of stresses and partial loss of composite action. At a deflection of 72.9 mm, a significant drop in load occurs, accompanied by extensive fracture and the propagation of splitting cracks in the timber. This results in a sudden reduction in strain in most reinforcement components, reflecting loss of load-carrying capacity and bond degradation. The compressive NSM bar exhibits only a minor reduction in strain, indicating that the compression zone remains relatively intact during this stage. No rupture of the FRP sheets or the NSM bars was observed, confirming that the failure was governed by timber fracture rather than reinforcement rupture. However, fracture of the vertical FRP strips in the shear span was observed (strain data for these strips were not recorded), indicating their active contribution in resisting longitudinal splitting prior to failure.

Although local FRP debonding and timber splitting were observed experimentally, these mechanisms were not the governing failure modes of the strengthened beams. The debonding was mainly associated with longitudinal shear stresses at the U-strips, while the final failure occurred through timber fracture at the bottom tension zone without debonding of the bottom FRP sheet. Therefore, the model assumption provides a reasonable approximation for flexural capacity prediction. The predicted moment of resistance of the strengthened beam calculated using Equation (24) is 30.1 kN·m, corresponding to a load capacity of 86.0 kN, which is 23% lower than the experimental value of 119.7 kN. The conservative prediction may be attributed to the idealized assumptions adopted in the analytical model, including perfect bond conditions, a simplified strain distribution, and the neglect of local interfacial effects, such as stress redistribution, partial composite action, and confinement provided by the hybrid FRP configuration. Future work should incorporate bond–slip behavior, interfacial damage, and stress concentration effects around NSM grooves to further improve the prediction of the failure process.

The effectiveness of the proposed hybrid strengthening system is attributed to the interaction among the NSM reinforcement, externally bonded FRP sheet, and U-strips, rather than the individual contributions of each component. The externally bonded FRP sheet helps protect the bottom NSM reinforcement, which experiences the highest tensile demand, against premature debonding, while the U-strips improve resistance against longitudinal shear, delay debonding of the externally bonded FRP, and protect the remaining NSM reinforcement. Therefore, the hybrid configuration promotes better strain compatibility and load sharing between the strengthening components. Although the combined strengthening effect does not necessarily represent a classical synergy where the capacity increase exceeds the sum of individual contributions, the hybrid system provides enhanced performance by preventing premature failure mechanisms that may limit the effectiveness of individual reinforcement systems. The advantage of the proposed approach, therefore, lies in improved composite action and failure-mode control rather than an additive increase in strength alone.

Although GFRP reinforcement enhances the flexural capacity of timber beams, FRP rupture is unlikely to govern because the tensile strength and stiffness of GFRP are considerably higher than those of timber. Increasing FRP thickness improves tensile resistance; however, it may shift the failure mode towards timber-controlled mechanisms, such as compression crushing in the compression zone, longitudinal shear failure (splitting parallel to the grain), or FRP–timber debonding. Therefore, the optimum FRP thickness should be selected by considering the interaction between flexural capacity, timber compression and shear resistance, and bond performance rather than maximizing the amount of reinforcement.

## 6. Conclusions

This research examined the flexural performance of timber beams reinforced with a hybrid strengthening technique consisting of near-surface-mounted (NSM) GFRP bars and externally bonded GFRP sheets through both experimental testing and analytical modeling. A generalized analytical formulation was developed for predicting the flexural strength of strengthened beams. Based on the experimental observations, constitutive modeling, and analytical validation, the following conclusions can be drawn:

- A comprehensive review and critical assessment of previous studies on strengthened timber beams were conducted, with particular emphasis on hybrid strengthening techniques involving NSM reinforcement and externally bonded FRP systems.
- A comprehensive analytical model was established to estimate the flexural capacity of timber beams reinforced using various combinations of NSM and externally bonded FRP systems. Based on the principles of internal force equilibrium and strain compatibility, the proposed formulation demonstrated satisfactory predictive accuracy, with discrepancies between calculated and experimental flexural strengths generally ranging from 2% to 23%. The proposed formulation employed a bilinear compressive constitutive model with post-peak softening, which successfully captured the experimentally observed compressive behavior of timber parallel to the grain, whereas the model for simulating the tensile response of timber was linear elastic up to failure.
- The experimental findings revealed that the hybrid reinforcement approach, incorporating NSM GFRP bars with externally bonded GFRP sheets, markedly enhanced the flexural performance of the timber beams, resulting in an increase in load-carrying capacity of up to approximately 84% relative to the unstrengthened control specimen.
- The hybrid strengthening approach also provided combined benefits in terms of strength, stiffness, and ductility, indicating that the interaction between NSM GFRP bars and externally bonded GFRP sheets can yield a more efficient strengthening mechanism than conventional single-system FRP strengthening techniques.
- The study further demonstrated that the interaction between NSM bars and externally bonded FRP sheets can be effectively incorporated into a unified generalized formulation, making the proposed model suitable for practical design and assessment of strengthened timber beams.
- Overall, this research establishes a unified experimental–analytical framework for evaluating and predicting the flexural strength of timber beams strengthened with hybrid FRP systems, thereby contributing to improved and more reliable design methodologies for sustainable timber rehabilitation and retrofitting applications.
- The proposed analytical model provides conservative predictions but is based on simplifying assumptions that limit its accuracy. The model does not explicitly account for bond–slip behavior, local stress concentrations around NSM grooves, or interactions among different FRP strengthening components. In addition, the adhesive layer is assumed to have a negligible direct contribution to the global load-carrying capacity due to its small thickness, although it influences interfacial bonding, polymer–wood interactions, curing behavior, and stress transfer. Future studies should incorporate these mechanisms to improve prediction accuracy and provide a more comprehensive understanding of the strengthening and failure behavior of hybrid NSM-FRP timber beams.

## Figures and Tables

**Table 1:** Force components and their distance from the top fibers for the strengthened timber beam’s section analysis.

Force Components (Figure 1)	Equations Nos.	Distance of the Force from Top Fiber (Figure 1)	Equations Nos.
$C_{1}=\frac{1}{2}byf_{wc}$	(1a)	$y_{c1}=c-\frac{2}{3}y$	(1b)
			
$C_{2}=\frac{1}{2}\left(1+\alpha \right)\left(c-y\right)bf_{wc}$	(2a)	$y_{c2}=\frac{\left(\alpha +2\right)}{3\left(\alpha +1\right)}\left(c-y\right)$	(2b)
			
$C_{3}=\varepsilon_{rc1}E_{r}\rho_{rc1}bh$	(3a)	$y_{c3}=y_{rc1}$	(3b)
			
$C_{4}=\varepsilon_{rc2}E_{r}\rho_{rc2}bh$	(4a)	$y_{c4}=y_{rc2}$	(4b)
			
$T_{1}=\frac{1}{2}f_{wt}b\left(h-c\right)$	(5a)	$y_{t1}=c+\frac{2}{3}\left(h-c\right)$	(5b)
			
$T_{2}=\varepsilon_{rt1}E_{r}\rho_{rt1}bh$	(6a)	$y_{t2}=h-y_{rt1}$	(6b)
			
$T_{3}=\varepsilon_{rt2}E_{r}\rho_{rt2}bh$	(7a)	$y_{t3}=h-y_{rt2}$	(7b)
			
$T_{4}=\varepsilon_{wt}E_{f}A_{f}$	(8a)	$y_{t4}=h$	(8b)

## Data Availability

The original contributions presented in this study are included in the article. Further inquiries can be directed to the corresponding author.

## References

[1] Stanila O., Isopescu D., Hohan R. (2010). Timber elements: Traditional and modern strengthening techniques. Bull. Polytech. Inst. Iași. Constr. Archit. Sect..

[2] Al-Mashgari H., Liu X., Nguyen T., Ngo T. (2024). Performance, methodology and opportunities in FRP strengthening techniques for timber structures: A state-of-the-art review. J. Build. Eng..

[3] Saad K., Lengyel A. (2022). Strengthening Timber Structural Members with CFRP and GFRP: A State-of-the-Art Review. Polymers.

[4] Frihart C.R., Rowell R. (2013). Wood Adhesion and Adhesives. Handbook of Wood Chemistry and Wood Composites.

[5] Custódio J., Broughton J., Cruz H. (2009). A review of factors influencing the durability of structural bonded timber joints. Int. J. Adhes. Adhes..

[6] Raftery G.M., Harte A.M., Rodd P.D. (2009). Bonding of FRP materials to wood using thin epoxy gluelines. Int. J. Adhes. Adhes..

[7] Liu Y., Fan J., Yao F., Gao X., Zhao Y., Liu B., Zhang Y., Li Y. (2024). Epoxy-acrylic polymer in-situ filling cell lumen and bonding cell wall for wood reinforcement and stabilization. Polymers.

[8] Triantafillou T.C., Deskovic N. (1991). Innovative prestressing with FRP sheets: Mechanics of short-term behavior. J. Eng. Mech..

[9] Fiorelli J., Dias A.A. (2003). Analysis of the strength and stiffness of timber beams reinforced with carbon fiber and glass fiber. Mater. Res..

[10] André A., Kliger R. Strengthening of timber beams using FRP, with emphasis on compression strength: A state of the art review. Proceedings of the Second Official International Conference of International Institute for FRP in Construction for Asia-Pacific Region APFIS.

[11] Nadir Y., Nagarajan P., Ameen M. (2016). Flexural stiffness and strength enhancement of horizontally glued laminated wood beams with GFRP and CFRP composite sheets. Constr. Build. Mater..

[12] Gilfillan J.R., Gilbert S.G., Patrick G.R.H. (2003). The use of FRP composites in enhancing the structural behavior of timber beams. J. Reinf. Plast. Compos..

[13] Yang Y.L., Liu J.W., Xiong G.J. (2013). Flexural behavior of wood beams strengthened with HFRP. Constr. Build. Mater..

[14] Vahedian A., Shrestha R., Crews K. (2019). Experimental and analytical investigation on CFRP strengthened glulam laminated timber beams: Full-scale experiments. Compos. Part B Eng..

[15] Jian B., Cheng K., Li H., Ashraf M., Zheng X., Dauletbek A., Zhou K. (2022). A review on strengthening of timber beams using fiber reinforced polymers. J. Renew. Mater..

[16] Schober K.U., Harte A.M., Kliger R., Jockwer R., Xu Q., Chen J.F. (2015). FRP reinforcement of timber structures. Constr. Build. Mater..

[17] Kliger I.R., Haghani R., Brunner M., Harte A.M., Schober K.U. (2016). Wood-based beams strengthened with FRP laminates: Improved performance with pre-stressed systems. Eur. J. Wood Wood Prod..

[18] Lehmann M., Properzi M., Pichelin F., Triboulot P. Pre-stressed FRP for the in-situ strengthening of timber structures. Proceedings of the 9th World Conference on Timber Engineering 2006 (WCTE 2006).

[19] Brady J.F., Harte A.M., Arima T. Prestressed FRP flexural strengthening of softwood glue-laminated timber beams. Proceedings of the World Conference on Timber Engineering (WCTE 2008).

[20] De Lorenzis L., Teng J.G. (2007). Near-surface mounted FRP reinforcement: An emerging technique for strengthening structures. Compos. Part B Eng..

[21] Gentile C., Svecova D., Rizkalla S.H. (2002). Timber beams strengthened with GFRP bars: Development and applications. J. Compos. Constr..

[22] Borri A., Corradi M., Grazini A. (2005). A method for flexural reinforcement of old wood beams with CFRP materials. Compos. Part B Eng..

[23] Lu W., Ling Z., Geng Q., Liu W., Yang H., Yue K. (2015). Study on flexural behaviour of glulam beams reinforced by Near Surface Mounted (NSM) CFRP laminates. Constr. Build. Mater..

[24] Yeboah D., Gkantou M. (2021). Investigation of flexural behaviour of structural timber beams strengthened with NSM basalt and glass FRP bars. Structures.

[25] Aslam M., Jin Y., Shafigh P. (2025). Recent Development in the Use of FRP Rods for Timber Beam Strengthening in NSM Technique. Global Conference on Materials Science and Engineering.

[26] Corradi M., Borri A., Righetti L., Speranzini E. (2017). Uncertainty analysis of FRP reinforced timber beams. Compos. Part B Eng..

[27] Kim Y.J., Harries K.A. (2010). Modeling of timber beams strengthened with various CFRP composites. Eng. Struct..

[28] Khelifa M., Celzard A. (2014). Numerical analysis of flexural strengthening of timber beams reinforced with CFRP strips. Compos. Struct..

[29] Saad K., Lengyel A. (2022). Inverse determination of material properties of timber beams reinforced with CFRP using the classical beam theory. Építőanyag-J. Silic. Based Compos. Mater..

[30] Hoseinpour H., Valluzzi M.R., Garbin E., Panizza M. (2018). Analytical investigation of timber beams strengthened with composite materials. Constr. Build. Mater..

[31] Ling Z., Liu W., Shao J. (2020). Experimental and theoretical investigation on shear behaviour of small-scale timber beams strengthened with Fiber-Reinforced Polymer composites. Compos. Struct..

[32] Malhotra S.K., Bazan I.M.M. (1980). Ultimate bending strength theory for timber beams. Wood Sci..

[33] (2025). Standard Test Methods for Direct Moisture Content Measurement of Wood and Wood-Based Materials.

[34] (2021). Standard Test Methods for Density and Specific Gravity (Relative Density) of Wood and Wood-Based Materials.

[35] (2025). Standard Test Methods for Small Clear Specimens of Timber.

[36] Lu P., Gilbert B.P., Kumar C., McGavin R.L., Karampour H. (2024). Influence of the moisture content on the fracture energy and tensile strength of hardwood spotted gum sawn timber and adhesive bonds (gluelines). Eur. J. Wood Wood Prod..

[37] Neely S.T. (1898). Relation of compression-endwise to breaking load of beam. Progress in Timber Physics.

[38] Glos P. (1978). Reliability Theory for Timber Structures: Determination of Compression Strength Behavior of Glulam Components from Interaction of Material Properties. Ph.D. Thesis.

[39] United States Department of Agriculture (USDA) Forest Service, Forest Products Laboratory (2010). Wood Handbook: Wood as an Engineering Material.

[40] Thelandersson S., Larsen H.J. (2003). Timber Engineering.

[41] (2004). Eurocode 5—Design of Timber Structures—Part 1-1: General Rules and Rules for Buildings.

